# Lactate dehydrogenase to albumin ratio as a prognostic indicator for patients with resectable non-small cell lung cancer

**DOI:** 10.1097/MS9.0000000000003705

**Published:** 2025-11-11

**Authors:** Bohua Wei, Weijie Li, Kun Qian, Peilong Zhang, Yuanbo Li, Kejian Shi, Yi Zhang

**Affiliations:** Department of Thoracic Surgery, Xuanwu Hospital, Capital Medical University, Beijing, China

**Keywords:** disease-free survival, lactate dehydrogenase to albumin ratio, non-small cell lung cancer

## Abstract

**Background:**

Serum lactate dehydrogenase to albumin ratio (LAR) is a newly discovered indicator that can comprehensively reflect the inflammatory or nutritional status of the body and has been proven to be associated with several diseases. This study aimed to investigate whether a high baseline LAR independently predicts worse prognosis in non-small cell lung cancer (NSCLC) patients undergoing radical surgery.

**Methods:**

This study retrospectively reviewed NSCLC patients who received radical surgery between January 2019 and October 2021 in our institution. Then, these patients were further categorized into low LAR and high LAR groups using X-tile software. Both univariate and multivariate logistic regression analyses were utilized to identify independent predictors of disease-free survival (DFS). A paired *t*-test was conducted to evaluate the changes in LAR after surgery.

**Results:**

A total of 233 patients were enrolled in this study. Patients with high baseline LAR had significantly worse DFS (*P* < 0.001) than those with low baseline LAR. According to Cox regression analysis, baseline LAR [hazard ratio (HR) 2.25, 95% confidence interval (CI) 1.01–5.01, *P* = 0.046] and tumor staging (HR 0.15, 95% CI 0.07–0.33, *P* < 0.001) were identified as independent predictors for DFS. Besides, based on the data of 97 patients who underwent their first follow-up at our hospital, cases with stage II/III tumors showed a significant decrease in LAR after surgery (*P* < 0.05), whereas no significant change was observed in stage I patients (*P* = 0.23).

**Conclusion:**

LAR emerged as a potential prognostic indicator for operable NSCLC patients. Cases with higher baseline LAR levels tend to have a poorer prognosis.

## Introduction

This article was compliant with the TITAN Guidelines 2025^[1]^. Currently, lung cancer is the most frequently diagnosed malignant tumor and the leading cause of cancer-related death worldwide^[2]^. It can be roughly classified as non-small cell lung cancer (NSCLC) and small cell lung cancer (SCLC) and NSCLC accounts for most of all cases. To date, surgery remains the only potentially curative modality for NSCLC and remains the preferred treatment for resectable early or middle-stage patients^[3]^. Although sub-lobar resection, especially segmentectomy, has been shown to achieve similar effects in recent years, lobectomy remains the standard of care resection strategy for operable patients^[4,5]^. However, many patients still experience recurrence and die from the disease despite radical resection^[6]^. Therefore, identifying easily accessible and cost-effective biomarkers to evaluate the risk of recurrence after surgery is of great value.

**HIGHLIGHTS**

- The first retrospective study to explore the potential value of baseline lactate dehydrogenase to albumin ratio (LAR) in resectable non-small cell lung cancer (NSCLC).
- Patients with stage II/III diseases showed a significant decrease in LAR after surgery, while stage I patients did not.
- Baseline LAR ≥ 5.4 was found to be closely associated with disease-free survival in operable NSCLC patients and can provide guidance for postoperative treatment strategy.

Glycolysis is one of the pivotal processes that fulfill the basic energy demands of cells. Lactate dehydrogenase (LDH) is one of the main enzymes involved in it, which accounts for catalyzing the transformation of lactate from pyruvate^[7]^. The level of serum LDH is a common biomarker used in clinical practice. An increased concentration may indicate several pathological conditions such as rhabdomyolysis, myocardial infarction, rheumatoid arthritis, pneumonia, and tumors^[7]^. Serum albumin (ALB) is a simple indicator to reflect the general nutritional status of the body. Considering that the growth of tumor cells requires a large amount of nutrients, a larger tumor burden often leads to hypoalbuminemia. Thus, a decreased level of serum ALB may imply a poor prognosis among patients with tumors^[8]^. More recently, lactate dehydrogenase to albumin ratio (LAR), which combines the two above-mentioned indicators together, was developed. It has been proven to be closely related to the prognosis of tumors, strokes, and infectious diseases^[9–11]^. However, there are limited data on its application in resectable NSCLC. Therefore, this study aimed to investigate whether a high baseline LAR independently predicts worse prognosis in NSCLC patients undergoing radical surgery.

## Methods

### Literature selection process

We conducted a comprehensive literature search on PubMed using keywords such as “lactate dehydrogenase to albumin ratio,” “prognosis,” and “non-small cell lung cancer.” Studies reporting associations between LAR and clinical outcomes in cancer patients were included and carefully reviewed to contextualize our research and identify gaps, particularly in resectable NSCLC.

### Patients

Ethical approval for this study (KS2024413) was provided by the Ethical Committee of our hospital on 1 November 2024.

Patients who underwent lobectomy and were pathologically confirmed to have NSCLC from January 2019 and October 2021 were included in this study. The specific inclusion criteria for the present study were as follows: (1) received radical lobectomy with systemic lymph node dissection; (2) pathologically confirmed as primary NSCLC; (3) exclude distant metastasis by positron emission tomography/computed tomography (CT) or brain magnetic resonance imaging (MRI), abdominal CT combined with whole body bone imaging before operation; and (4) had complete serum LDH and ALB data within 5 days before operation. We excluded patients who (1) had incomplete data; (2) received neoadjuvant chemotherapy, immunotherapy, targeted therapy, or radio therapy before surgery; (3) had a history of other surgery, severe trauma, stroke, myocardial infarction, infectious diseases, or rhabdomyolysis within 6 months prior to operation; (4) had a history of other malignancies; (5) received intravenous nutritional support treatment within 1 month before surgery; and (6) were lost to follow-up.

### Data collection

In general, data collection was conducted through the inpatient medical record system and telephone follow-up.

Clinical characteristics, including age, sex, body mass index [calculated as weight (in kg)/height^2^ (in m2)], smoking history, comorbidities, tumor location, and surgical procedures, were collected. Pathological features included pathological type, visceral pleural invasion (VPI), spread through air spaces (STAS), and lymphovascular invasion were collected. Tumor staging was determined according to the ninth edition TNM classification of lung cancer based on pathological results^[12]^.

For LDH and ALB, all blood samples were drawn within 5 days before surgery in a fasting state at 5 o’clock in the morning and were transferred to the lab within 2 hours. The concentrations of LDH and ALB were measured by the central laboratory of our hospital. LAR was calculated as the ratio of serum LDH (IU/L) to ALB (g/L). Postoperative LAR was obtained approximately 3 months after surgery during the first follow-up at our hospital. Among the 233 enrolled patients, a total of 97 patients underwent their first follow-up examination at our hospital and obtained their postoperative LAR data, which were used to explore changes in LAR after surgery.

The follow-up strategy was determined based on postoperative time through outpatient medical records or by telephone. The specific standards were once every 3 months within 1 year after surgery; once every 6 months between 1 and 3 years; and once every year for more than 3 years if there are no signs of recurrence or metastasis in the previous reexamination. The items for re-examination include routine blood test, blood biochemical test, blood tumor markers, chest CT scan, abdominal ultrasonography, cranial MRI, and whole-body bone scan. The specific follow-up time points and items are shown in Supplemental Digital Content Table S1, available at: http://links.lww.com/MS9/B15. The last follow-up date was 31 October 2024, ensuring a minimum follow-up period of 3 years for most patients who underwent re-examination as required. Disease-free survival (DFS) was computed as the time from surgery to the last follow-up or tumor recurrence or metastasis.

### Optimal cutoff value and groups

The optimal cutoff value of LAR was identified using X-tile software^[13]^. Briefly, the software used different values of LAR as potential cutoff values to divide the patients into different groups. Then, the software performed log-rank tests to calculate *P*-values for each potential cutoff value. Finally, the software selected the cutoff value that minimizes the *P*-value as the optimal cutoff value. In the current study, the optimal cutoff value for baseline LAR was 5.4. Subsequently, patients with baseline LAR ≥ 5.4 were divided into the high LAR group, while those with baseline LAR < 5.4 were assigned to the low LAR group.

### Statistical analysis

The statistical software IBM SPSS (version 26.0) and R software (version 4.4.1) were utilized for statistical analysis. We represented continuous variables as mean ± standard deviation (SD) or medians [interquartile ranges (IQRs)] based on whether the distribution was normal. Then Student’s *t*-test or the Mann–Whitney *U* test was used for comparing the differences between the groups. Categorical variables were presented as numbers and percentages and were analyzed with the chi-square test or Fisher’s exact test. A paired *t*-test was used to evaluate the changes in LAR after surgery. Kaplan–Meier curves with Log-rank test were applied to assess DFS based on tumor stage or LAR. Censoring was handled according to standard survival analysis methods. Patients who were lost to follow-up or did not experience the event of interest were censored at the time of their last follow-up. Univariate and multivariate Cox regression analyses were conducted, and hazard ratios (HRs) with 95% confidence intervals (CIs) were computed to pinpoint the predictors of DFS. Calibration curves with the Hosmer–Lemeshow (HL) test at three time points were drawn to evaluate the accuracy of prognosis prediction. We determined a two-tailed *P*-value of less than 0.05 to be statistically significant.

## Results

**Figure 1.**
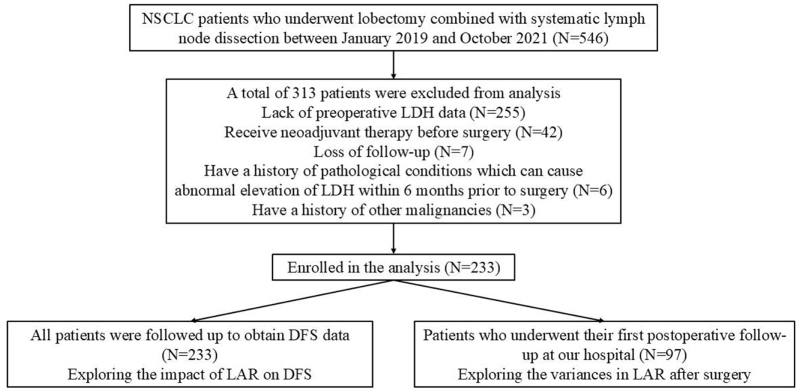
The flow chart of the study.

A total of 546 patients received lobectomy with systemic mediastinal lymph node dissection due to NSCLC in our institution from January 2019 to October 2021. However, a total of 313 cases were excluded mainly due to the lack of preoperative serum LDH data, as well as neoadjuvant therapy, recent surgeries, pneumonia, rheumatoid arthritis, history of other malignancies, or loss of follow-up. Therefore, the remaining 233 patients were finally enrolled in the study. The specific flow chart of the study is shown in Figure 1.

**Figure 2.**
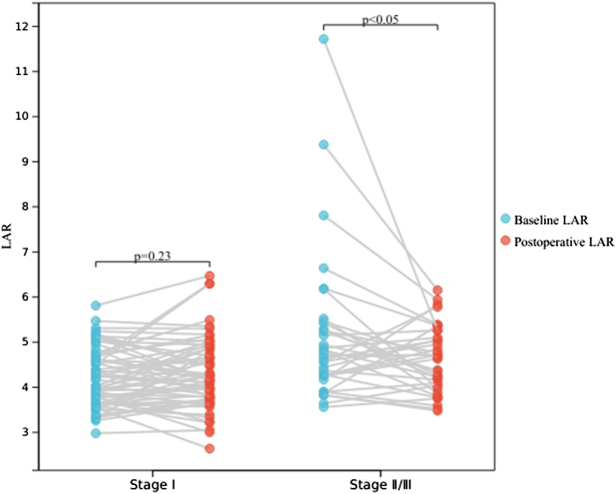
Changes in LAR after surgery in patients with different stages of disease.

According to the ninth edition TNM classification of lung cancer, a total of 167 patients were confirmed as having stage I disease, while the other 66 patients were evaluated as having stage II or III tumors (including 47 with stage II and 19 with stage III). Pathological types include 197 cases of lung adenocarcinoma (LUAD), 24 cases of lung squamous cell carcinoma (LUSC), 5 cases of adenosquamous carcinoma, 3 cases of carcinosarcoma, 3 cases of carcinoid, and 1 case of pleomorphic carcinoma. The entire cohort had a median follow-up time of 39 months (range, 12–69 months), during which a total of 40 patients developed recurrence or metastasis. The clinicopathological features of all enrolled patients and the comparison between stage I and stage II/III cases are presented in Table 1. Those with relatively late-stage diseases tended to be male and smokers. Regarding LAR, stage I patients had significantly lower baseline LAR than stage II/III cases. In terms of pathological features, stage II/III patients had a higher incidence of pleural invasion and lymphovascular invasion, while there was no significant difference in the incidence of STAS. Besides, it was easy to understand that the DFS of stage I patients was significantly longer than that of stage II/III patients. Subsequently, LAR from the first postoperative re-examination was analyzed. A total of 97 (62 stage I and 35 stage II/III) patients received their first post-operative follow-up at our hospital. As shown in Fig. 2, stage II/III patients showed a significant decrease in LAR after surgery (*P* < 0.05), while stage I patients did not (*P* = 0.23).

**Table 1:** The baseline characteristics and the comparison of patients with different tumor staging

Variables	Total (*n* = 233)	Stage I (*n* = 167)	Stage II/III (*n* = 66)	*P*-value
Age	61.18 ± 9.23	60.56 ± 9.28	62.77 ± 8.97	0.099
Sex				
Male	109 (46.8%)	68 (40.7%)	41 (62.1%)	0.003*
Female	124 (53.2%)	99 (59.3%)	25 (37.9%)	
BMI	24.52 ± 3.64	24.63 ± 3.61	24.22 ± 3.73	0.433
Smoking history				0.004*
Yes	83 (35.6%)	50 (29.9%)	33 (50.0%)	
No	150 (64.4%)	117 (70.1%)	33 (50.0%)	
Hypertension				0.250
Yes	39 (16.7%)	25 (15.0%)	14 (21.2%)	
No	194 (83.3%)	142 (85.0%)	52 (78.8%)	
Diabetes				0.792
Yes	19 (8.2%)	13 (7.8%)	6 (9.1%)	
No	214 (91.8%)	154 (92.2%)	60 (90.9%)	
Coronary heart disease				0.374
Yes	15 (6.4%)	9 (5.4%)	6 (9.1%)	
No	218 (93.6%)	158 (94.6%)	60 (90.9%)	
Chronic kidney disease				1.000
Yes	3 (1.3%)	2 (1.2%)	1 (1.5%)	
No	230 (98.7%)	165 (98.8%)	65 (98.5%)	
Tumor location				0.888
Left	105 (45.1%)	77 (46.1%)	28 (42.4%)	
Right	128 (54.9%)	90 (53.9%)	38 (57.6%)	
Baseline LAR	4.47 [3.92,4.98]	4.41 [3.79,4.85]	4.66 [4.17,5.32]	0.001*
Pleural invasion				0.003*
Yes	88 (37.8%)	53 (31.7%)	35 (53.0%)	
No	145 (62.2%)	114 (68.3%)	31 (47.0%)	
Lymphovascular invasion				0.008*
Yes	11 (4.7%)	4 (2.4%)	7 (10.6%)	
No	222 (95.3%)	163 (97.6%)	59 (89.4%)	
STAS				0.120
Yes	10 (4.3%)	5 (3.0%)	5 (8.2%)	
No	223 (95.7%)	162 (97.0%)	61 (91.8%)	
Pathological type				<0.001*
LUAD	197 (84.5%)	157 (94.0%)	40 (60.6%)	
LUSC	24 (10.3%)	8 (4.8%)	16 (24.2%)	
Others	12 (5.2%)	2 (1.2%)	10 (15.2%)	
DFS	41 [36,49]	44 [37,50]	36.5 [19.75, 45]	<0.001*

BMI, body mass index; LAR, lactate dehydrogenase to albumin ratio; STAS, spread through air spaces; LUAD, lung adenocarcinoma; LUSC, lung squamous cell carcinoma; DFS, disease-free survival.

^*^ Statistically significant (*P*-value < 0.05).

**Figure 3.**
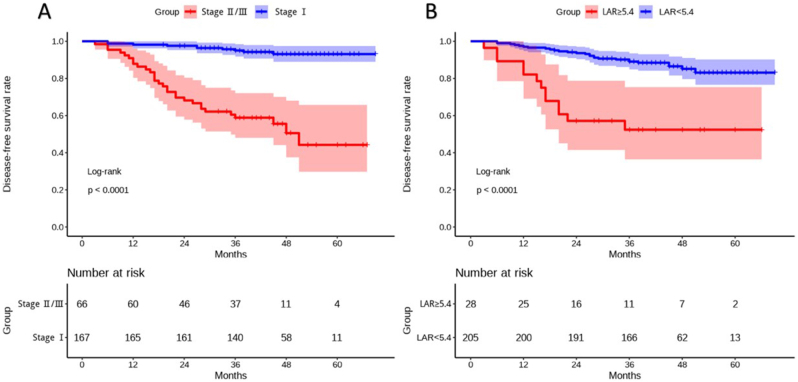
(A) Disease-free survival (DFS) of patients with different stages of disease; (B) DFS of patients with different baseline LAR levels.

Next, we determined the optimal cutoff value for baseline LAR as 5.4 using X-tile software based on DFS data. Thereupon, patients were divided into the high LAR and low LAR groups. As shown in Table 2, patients with high baseline LAR were older, had a higher proportion of males, and had a higher rate of smoking history. Besides, their proportion of LUAD was relatively low, while the proportion of LUSC or other types was high. More importantly, patients with high baseline LAR had a relatively late stage, resulting in significantly worse DFS compared with cases with low baseline LAR (*P* < 0.001). Then we depicted Kaplan–Meier curves based on tumor staging and baseline LAR to visually describe patients’ DFS using R software with “survminer” packages (Fig. 3). Patients with early staging diseases or lower baseline LAR had significantly better prognosis (*P* < 0.0001). Afterward, we investigated whether baseline LAR was an independent predictor of DFS. As shown in Table 3, sex, smoking history, baseline LAR, pleural invasion, pathological type, and tumor staging were all confirmed to be associated with DFS through univariate Cox regression analysis. However, only high baseline LAR (HR 2.25, 95% CI 1.01–5.01, *P* = 0.046) and early tumor staging (HR 0.146, 95% CI 0.07–0.33, *P* < 0.001) were identified as independent predictors of prognosis based on multivariate Cox analysis. Subsequently, considering the significant differences in sex, smoking history, and disease stage between patients in the high-LAR and low-LAR groups, R software with “WeightIt” and “cobalt” packages was used to perform inverse probability weighting to balance these confounding factors. Post-weighting, all standardized mean differences were <0.1, indicating adequate covariate balance (Supplemental Digital Content Figure S1, available at: http://links.lww.com/MS9/B14). Subsequent Cox proportional hazards regression analysis using the weighted data continued to show that LAR ≥5.4 remained an independent risk factor (Supplemental Digital Content Table S2, available at: http://links.lww.com/MS9/B16). Finally, the calibration curves at 12-month, 36-month, and 60-month time points were plotted, with HL tests indicating *P*-values all higher than 0.05, demonstrating the model’s good predictive capability (Supplemental Digital Content Figure S2, available at: http://links.lww.com/MS9/B14).

**Table 2:** Clinicopathological characteristics of enrolled patients stratified by baseline LAR

Variables	LAR ≥ 5.4 (*n* = 28)	LAR < 5.4 (*n* = 205)	*P*-value
Age	65.77 ± 7.52	60.56 ± 9.28	0.005*
Sex			0.017*
Male	19 (67.8%)	90 (43.5%)	
Female	9 (32.2%)	115 (56.5%)	
BMI	24.46 ± 2.38	24.52 ± 3.78	0.929
Smoking history			0.003*
Yes	17 (60.7%)	66 (32.2%)	
No	11 (39.3%)	139 (67.8%)	
Hypertension			0.277
Yes	7 (25.0%)	32 (15.6%)	
No	21 (75.0%)	173 (84.4%)	
Diabetes			0.710
Yes	3 (10.7%)	16 (7.8%)	
No	25 (89.3%)	189 (92.2%)	
Coronary heart disease			0.400
Yes	3 (10.7%)	12 (5.9%)	
No	25 (89.3%)	193 (94.1%)	
Chronic kidney disease			0.320
Yes	1 (3.6%)	2 (1.0%)	
No	27 (96.4%)	203 (99.0%)	
Tumor location			0.802
Left	12 (42.9%)	93 (45.4%)	
Right	16 (57.1%)	112 (54.6%)	
Pleural invasion			0.066
Yes	15 (53.6%)	73 (35.6%)	
No	13 (46.4%)	132 (64.4%)	
Lymphovascular invasion			0.519
Yes	2 (7.1%)	9 (4.4%)	
No	26 (92.9%)	196 (95.6%)	
STAS			0.841
Yes	1 (3.6%)	9 (4.4%)	
No	27 (96.4%)	196 (95.6%)	
Pathological type			0.005*
LUAD	18 (64.3%)	179 (87.3%)	
LUSC	6 (21.4%)	18 (8.8%)	
Others	4 (14.3%)	8 (3.9%)	
Tumor staging			<0.001*
I	12 (42.9%)	155 (75.6%)	
II/III	16 (57.1%)	50 (24.4%)	
DFS	28.5 [16.25, 46.5]	42 [37,49]	<0.001*

BMI, body mass index; DFS, disease-free survival; LAR, lactate dehydrogenase to albumin ratio; LUAD, lung adenocarcinoma; LUSC, lung squamous cell carcinoma; STAS, spread through air spaces.

^*^ Statistically significant (*P*-value < 0.05).

**Table 3:** Univariate and multivariate Cox regression analyses for indicators associated with DFS

Variables	Univariate		Multivariate		
	HR (95% CI)	*P*-value		HR (95% CI)	*P*-value
Age	1.03 (0.99–1.07)	0.102			
Sex		0.017*			0.942
Male	2.22 (1.15–4.27)			0.96 (0.34–2.76)	
Female	Ref			Ref	
BMI	0.96 (0.89–1.04)	0.344			
Smoking history		0.006*			0.468
Yes	2.40 (1.28–4.51)			1.48 (0.51–4.29)	
No	Ref			Ref	
Hypertension					
Yes	1.65 (0.81–3.39)	0.169			
No	Ref				
Diabetes					
Yes	0.83 (0.25–2.69)	0.750			
No	Ref				
Coronary heart disease					
Yes	1.77 (0.63–4.97)	0.280			
No	Ref				
Chronic kidney disease					
Yes	0.05 (0.00–3421.88)	0.595			
No	Ref				
Tumor location		0.706			
Left	Ref				
Right	0.89 (0.48–1.65)				
Baseline LAR		<0.001*			0.046*
≥5.4	4.88 (2.51–9.48)			2.25 (1.01–5.01)	
<5.4	Ref			Ref	
Pleural invasion		0.004*			0.209
Yes	2.54 (1.36–4.76)			1.54 (0.79–3.01)	
No	Ref			Ref	
Lymphovascular invasion		0.064			
Yes	2.65 (0.94–7.64)				
No	Ref				
STAS		0.243			
Yes	2.02 (0.62–6.55)				
No	Ref				
Pathological type		<0.001*			0.564
LUAD	Ref			Ref	
LUSC	2.45 (1.07–5.62)	0.035*		0.69 (0.28–1.71)	0.425
Others	6.75 (2.78–16.42)	<0.001*		1.26 (0.48–3.34)	0.640
Tumor staging		<0.001*			<0.001*
I	0.10 (0.05–0.21)			0.15 (0.07–0.33)	
II/III	Ref			Ref	

BMI, body mass index; CI, confidence interval; DFS, disease-free survival; HR, hazard ratio; LAR, lactate dehydrogenase to albumin ratio; LUAD, lung adenocarcinoma; LUSC, lung squamous cell carcinoma; STAS, spread through air spaces.

^*^ Statistically significant (*P*-value < 0.05).

## Discussion

In the current study, we found that LAR significantly decreased after surgery in stage II/III NSCLC patients, whereas no significant change was observed in stage I cases. This suggests that LAR is positively correlated with tumor burden and can be reduced by tumor resection. Notably, multivariate Cox regression analysis revealed that elevated baseline LAR independently predicted inferior DFS for operable NSCLC patients. Therefore, it can serve as a simple but effective indicator for identifying high-risk patients prone to postoperative recurrence in NSCLC. By integrating LAR with other prognostic factors, such as tumor staging, clinicians can make personalized postoperative management decisions regarding adjuvant therapy and follow-up strategies. For instance, patients with high baseline LAR and advanced tumor staging may benefit from more aggressive adjuvant therapy or closer follow-up.

LDH, the enzyme catalyzing pyruvate conversion to lactate during glycolysis, is predominantly intracellular. When cells suffer damage such as that inflicted by high tumor burden, LDH will be released into the circulation, explaining the correlation between tissue damage and elevated serum LDH levels^[14]^. Critically, elevated tumor LDH production reflects increased glycolytic flux. The resulting lactate accumulation acidifies the tumor microenvironment (TME), directly suppressing cytotoxic T lymphocyte and natural killer cell function while promoting the recruitment and activity of immunosuppressive cells like regulatory T cells (Tregs) and myeloid-derived suppressor cells^[15]^. Thus, high serum LDH signifies not only tumor burden and damage but also an immunosuppressive TME associated with poor survival across multiple cancers^[16,17]^. Conversely, hypoalbuminemia is a hallmark of cancer cachexia. It results from hepatic reprioritization triggered by systemic inflammation during the acute-phase response^[18]^. Additionally, hypoalbuminemia indicates the severe malnutrition characteristic of cancer cachexia, which is driven by the tumor’s metabolic demands as well as cachexia-inducing factors. Therefore, LAR, which combines LDH and ALB, can comprehensively reflect the tumor burden and the overall metabolic and inflammatory status of patients. For instance, Hu and colleagues constructed a nomogram based on LAR, tumor staging, and other factors to predict the overall survival (OS) and DFS for colorectal cancer patients^[19]^. Another study confirmed that baseline LAR was closely related OS of bladder cancer patients^[20]^. As for NSCLC, elevated LAR was shown to be a useful prognostic predictor of patients with metastatic NSCLC treated with nivolumab recently^[21]^. But there is still limited research on its application in operable patients. As far as we know, this study is the first to explore the variations in LAR after surgery for NSCLC patients and their impact on prognosis.

As expected, baseline LAR was significantly higher in stage II/III patients compared to stage I cases. This likely reflects the larger tumor burden and more aggressive disease state associated with advanced stages. Consequently, a high baseline LAR signifies greater metabolic stress and tumor burden, ultimately leading to a poorer prognosis. While TNM staging remains the cornerstone of prognosis, LAR can provide additional information on the metabolic and inflammatory status of patients, which can assist in identifying cases with high recurrence risk in patients with the same stage. However, other factors, such as systemic chronic inflammation, age-related changes in metabolism, gender-specific hormonal influences, and the effects of smoking on LDH release and ALB synthesis, could also confound the comparison of LAR between early and relatively late-stage patients in the present study. Therefore, further studies are needed to eliminate the influence of these confounders.

Recently, a systematic review and meta-analysis summarized recurrence-free survival (RFS) in patients with surgically resected NSCLC, pointing out that the 5-year RFS ranges from 34% to 82% based on different tumor staging, which reveals that postoperative recurrence is quite common^[22]^. Therefore, identifying patients at high risk of recurrence is of great significance. Our study confirms that baseline LAR can serve as a simple but effective indicator for identifying high-risk patients for postoperative recurrence. In addition to conventional imaging and laboratory projects, circulating tumor DNA (ctDNA) minimal residual disease has been proven to be an effective method for monitoring the recurrence of NSCLC^[23]^. A recent multi-center study also found that ctDNA positivity after surgery preceded radiological recurrence by a median of 6.6 months^[24]^. But currently, its relatively expensive cost limits its widespread application in clinical practice. Conversely, LAR can be calculated from routine preoperative blood tests, making it practical for widespread clinical use. Therefore, comprehensively considering LAR and tumor staging to determine patients for ctDNA monitoring may be a good strategy.

VPI, STAS, and lymphovascular invasion are all common pathological features of poor prognosis after surgery, which do not influence general tumor staging^[25,26]^. Adjuvant therapy is more commonly recommended when these risk factors exist, especially in controversial stage IB patients^[4]^. However, these factors showed no significant impact on DFS, whereas baseline LAR emerged as an auxiliary biomarker for postoperative adjuvant therapy.

Except for LAR, there are also several simple and accessible hematological indicators that have been confirmed to be closely related to the prognosis of NSCLC patients. For example, in terms of peripheral blood inflammatory cell counts, pre-treatment neutrophil-to-lymphocyte ratio, platelet-to-lymphocyte ratio, monocyte-to-lymphocyte ratio, and systemic immune-inflammatory index have been reported to be closely related to the prognosis of NSCLC patients^[27,28]^. Regarding serum tumor markers, cytokeratin-19 fragment (CYFRA 21-1) and carcinoembryonic antigen have been proven to be prognostic indicators for NSCLC patients^[29,30]^. Additionally, in terms of coagulation-related indicators, preoperative fibrinogen and D-dimer levels can also be used as prognostic indices for NSCLC^[27,31]^. Therefore, combining LAR with these indicators may further enhance prognostic predictive efficacy.

Several limitations need to be pointed out in our study. First, this study’s retrospective design introduces inherent biases, including incomplete follow-up data and unmeasured confounders. Besides, challenges of retrospective data analysis include standardizing LAR measurement protocols and non-uniformity in follow-up data collection. Crucially, the single-center nature of this retrospective study significantly impacts its external validity. Our findings reflect the specific patient demographics, local clinical practices, and healthcare setting of our institution. Consequently, the prognostic performance and clinical applicability of the LAR identified in our cohort may not be directly transferable to populations from different geographic regions or healthcare systems. Therefore, prospective validation in diverse, multi-center cohorts representing varied populations and practice settings is critical to confirm LAR’s broader prognostic utility and generalizability. Next, not all patients received follow-up examinations at our hospital, so there might be bias in inquiring about recurrence through phone calls. In addition, the optimal cutoff value of baseline LAR obtained in the present study, derived from our specific patient population, may not be universally applicable. The optimal cutoff values determined by previous studies range from 3.8 to 12.3, with significant differences, underscoring the potential influence of population-specific factors and center effects^[9,18–20]^. Therefore, to robustly establish the generalizability of LAR as a prognostic biomarker and to define universally applicable or regionally tailored cutoff values, large-scale collaborative multi-center prospective clinical trials incorporating standardized protocols across different geographical regions are warranted in the future.

## Conclusions

In summary, our study confirmed that baseline LAR can serve as a potential prognostic indicator in NSCLC patients who have undergone radical surgery. Further research is needed to validate its prognostic value in larger cohorts and to explore its integration with other biomarkers.

## Data Availability

The datasets analyzed during the current study are available upon reasonable request.

## Research registration unique identifying number (UIN)

This research was registered on the National Medical Research Filing and Registration Platform (https://www.medicalresearch.org.cn/; Project Number: XWZC20220609).
